# Demographic and Clinical Characteristics of Migrant Patients Visiting the Emergency Department

**DOI:** 10.7759/cureus.39746

**Published:** 2023-05-30

**Authors:** Busra Bildik, Mert Aker

**Affiliations:** 1 Emergency Medicine, Karabuk Training and Research Hospital, Karabuk, TUR; 2 Cardiology, Karabuk Training and Research Hospital, Karabuk, TUR

**Keywords:** immigrant health, delivery of health care, refugee health, emigrants and immigrants, emergency medicine

## Abstract

Introduction

Disasters, war, violence, and famine have driven people to migrate in search of a better life, resulting in an increasing number of health issues related to migration. Turkiye has historically been a host country for migration due to its geopolitical location for economic and educational reasons, among other reasons. Migrants frequently visit emergency departments (EDs) regarding their chronic or acute diseases. Understanding the characteristics and admission diagnosis in EDs can help healthcare providers identify areas that require attention. This study aimed to determine the demographic characteristics and most frequent reasons for migrant patients visiting the ED.

Methods

This retrospective cross-sectional study was conducted in the ED of a tertiary hospital in Turkiye between January 1, 2021, and January 1, 2022. Sociodemographic data and diagnoses were obtained from the hospital information system and medical records. Migrant patients who visited the ED for any reason were included, while patients with inaccessible data, no diagnosis code, or missing information were excluded. Data were analyzed using descriptive statistical methods and compared using the Mann-Whitney U test, Student's t-test, and Chi-squared test.

Results

Out of 3865 migrant patients, 2186 (56.6%) were male, and the median age was 22 (17-27) years. Most patients (74.5%) were from the Middle East, and 16.6% were from Africa. The most common reasons for visiting the hospital were R00-99 "Symptoms, signs, and abnormal clinical and laboratory findings, not elsewhere classified" (45.6%); M00-99 "Diseases of the musculoskeletal system and connective tissue" (29.2%); and J00-99 "Diseases of the respiratory system" (23.1%). Among the African patients, 82.7% were students, while 85.4% of Middle Eastern patients were non-students. The number of visits differed significantly between regions, with Middle Easterners visiting more frequently than Africans and Europeans.

Conclusion

The majority of the patients were from the Middle East. Also, patients from the Middle East had more visits and a higher likelihood of being hospitalized than patients from other regions. The sociodemographic characteristics of migrant patients visiting ED and information about their diagnoses can help determine the profile of patients that emergency physicians are likely to encounter.

## Introduction

Disasters, war, violence, and famine force people to flee their countries to search for a better life; thus, the importance of migration and the resulting health problems continue to increase [1]. In particular, Turkiye has historically been recognized as a host country for migration owing to its geopolitical location [2]. Although war and famine were recognized as the driving forces of migration in the past, recent reports have indicated that people are also migrating from Asia, the Middle East, and Africa for economic reasons and education [3]. Educational migration is associated with health risks related to the young age of migrants, cultural and language differences, limited social connections, and a lack of social security [4]. Contact with healthcare services seems to be focused on infectious diseases among migrants from African countries where infectious diseases are more prevalent. However, it should be noted that some migrants also suffer from chronic diseases. Moreover, it has been reported that the lack of information about the prevalence of these diseases in the countries of origin affects the quality of healthcare services provided to immigrants [5]. When migrants seek healthcare, they most frequently visit emergency departments (EDs), regardless of their conditions or diseases [6,7]. Information about the demographic characteristics and the most common reasons for the ED use of migrants may provide insights into the types of services and help us to identify areas requiring particular attention. In this study, we aimed to determine the demographic characteristics of migrants visiting ED and the most common reasons for their visits to hospitals.

## Materials and methods

Study design

This retrospective cross-sectional study was conducted at the ED of a tertiary hospital between January 1, 2021, and January 1, 2022. Sociodemographic data and information about the diagnosis of the patients were retrieved from the medical records. This study was approved by the local ethics committee (Decision No: 2023/1285).

Selection of patients

The present study included migrant patients who visited the ED of our hospital for any reason. Conversely, patients whose data were not accessible from medical records and those missing data were excluded from the study.

Data collection

Patients' data, including age, sex, student status, country of origin, repeat visits (if any) and diagnosis code, number of visits, diagnosis code assigned at the time of the first visit, and clinical outcomes, were recorded.

Outcome

The study outcomes were the demographic characteristics of migrant patients visiting the ED and the most frequent reasons for visiting the hospital.

Statistical analysis

Statistical analysis of the study data was performed using the IBM SPSS Statistics 22 (IBM SPSS, Turkiye) program. Further, the study data were examined for the normality of distribution using the Kolmogorov-Smirnov and Shapiro-Wilk tests and assessed using descriptive statistical methods (presented as mean, standard deviation, median and interquartile ranges, and frequency). Non-normally distributed numerical data were analyzed using the Mann-Whitney U test, whereas normally distributed data were analyzed using the Student's t-test. Nominal data were compared using the chi-square test. Multiple groups were compared using posthoc analysis with p-value adjustment. Statistical significance was set at p < 0.05.

## Results

Overall, 3865 patients were included in the present study. The median age of the participants was 22 (17-27) years, and of 3865 participants, 2186 (56.6%) were male. Regarding the region of origin, 2880 (74.5%) participants were from the Middle East, and 640 (16.6%) participants were from Africa. Among the participants, 2733 (70.7%) were not students. The first three diagnosis codes that were found to be the most common reasons for patient visit the hospital were as follows: symptoms, signs, and abnormal clinical and laboratory findings, not elsewhere classified (R00-99) in 1763 (45.6%) patients; diseases of the musculoskeletal system and connective tissue (M00-99) in 1128 (29.2%) patients; and diseases of the respiratory system (J00-99) in 892 (23.1%) patients. Other demographic characteristics of the patients are presented in Table 1.

**Table 1:** Demographic characteristics, diagnosis codes, and number of visits

Variable		n (%), mean ± SD
Gender, age	Male	2186 (56.6), 22.0 ± 12.72
	Female	1679 (43.4), 24.37 ±15.53
Region of origin	Africa	640 (16.6)
	America	16 (0.4)
	Europe	27 (0.7)
	Asia	302 (7.8)
	Middle East	2880 (74.5)
Education status	Students	1132 (29.3)
	Nonstudents	2733 (70.7)
Social security	Yes	1104 (28.6)
	No	2761 (71.4)
Readmission	Yes	1731 (44.8)
	No	2134 (55.2)
Diagnosis codes	Certain infectious and parasitic diseases (A00-B99)	117(3)
	Neoplasms (C00-D48)	11(0.3)
	Diseases of the blood and blood-forming organs and certain disorders involving the immune mechanism (D50-D89)	15(0.4)
	Endocrine, nutritional and metabolic diseases (E00-E90)	14(0.4)
	Mental, Behavioral and Neurodevelopmental disorders (F00-F99)	44(1.1)
	Diseases of the nervous system (G00-G99)	62 (1.6)
	Diseases of the eye and adnexa (H00-H59)	124(3.2)
	Diseases of the ear and mastoid process (H60-H95)	112 (2.9)
	Diseases of the circulatory system (I00-I99)	82 (2.1)
	Diseases of the respiratory system (J00-J99)	892 (23.1)
	Diseases of the digestive system (K00-K93)	295 (7.6)
	Diseases of the skin and subcutaneous tissue (L00-L99)	175 (4.5)
	Diseases of the musculoskeletal system and connective tissue (M00-M99)	1128 (29.2)
	Diseases of the genitourinary system (N00-N99)	249 (6.4)
	Pregnancy, childbirth and the puerperium (O00-O99)	6 (0.2)
	Certain conditions originating in the perinatal period (P00-P96)	13 (0.3)
	Congenital malformations, deformations and chromosomal abnormalities (Q00-Q99)	1 (<0.1)
	Symptoms, signs, and abnormal clinical and laboratory findings, not elsewhere classified (R00-R99)	1763 (45.6)
	Injury, poisoning and certain other consequences of external causes (S00-T98)	438 (11.3)
	External causes of morbidity (U50-Y98)	18 (0.5)
	Factors influencing health status and contact with health services (Z00-Z99)	469 (12.1)
	Codes for special purposes (U00-U49)	1 (<0.1)
	Forensic examination (X)	73 (1.9)
Different diagnosis	Yes	1474 (38.1)
	No	2391 (61.9)
Clinical outcome	Inpatient care	3599 (93.1)
	Outpatient care	266 (6.9)
Age	Mean ± SD	23.03 ± 14.06
Number of visits	Mean ± SD	2.13 ± 2.08

There was a statistically significant difference between patients who were and were not students in terms of the region of origin (p<0.001). Notably, the posthoc analysis revealed a significant difference between migrants from Africa and those from other regions in terms of student status. Among the African patients, 529 (82.7%) were students. Among the Middle Eastern patients, 2460 (85.4) were not students; this number was significantly higher than that of patients from Africa and Asia, but it was not significantly different from that of patients from America and Asia (p<0.001 and p<0.001, respectively) (Table 2).

**Table 2:** Comparison between students and nonstudents in terms of demographic characteristics, diagnosis codes, and number of visits ¥ Pearson Chi-squared test was used

Parameter	Students	Nonstudents	Total	p-value	
Age Mean ± SD	22.19 ± 3.75	23.38 ± 16.53	23.03 ± 14.06	0.919	
Gender (female) n (%)^¥^	381 (33.7)	1298 (47.5)	1679 (100)	0.001*	
Number of visits Median (Q1-Q3)	1 (1-2)	2 (1-3)	1 (1-2)	0.001*	
Clinical outcome n (%)					
Outpatient care	1099 (30.5)	2500 (69.5)	3599 (100)	0.001*	
Inpatient care	33 (12.4)	233 (87.6)	266 (100)		
Region of origin n (%)					
Africa^1^	529 (82.7)	111 (17.3)	640	<0.001*	p^1-2^= 0.001*
					p^1-3^= 0.001*
America^2^	4 (25)	12 (75)	16		p^1-4^= 0.001*
					p^1-5^=0.001*
Europe^3^	3 (11.1)	24 (88.9)	27		p^2-3^=0.394
					p^2-4 ^=0.009
Asia^4^	176 (58.3)	126 (41.7)	302		p^2-5^=0.240
					p^3-4^=0.001*
Middle East^5^	420 (14.6)	2460 (85.4)	2880		p^3-5^=0.611
					p^4-5^=0.001*

There was a statistically significant relationship between the number of presentations and the region of origin. According to the posthoc analysis, this difference was caused by the differences between Middle Easterners and Africans, Middle Easterners and Europeans, and Middle Easterners and Asians (p<0.001, p<0.001, and p<0.001, respectively). The median number of visits for patients from the Middle East was 2 (1-3), which was higher than that for the other three groups. However, there was no statistically significant difference between patients from the Middle East and America in terms of the number of visits (p=0.145) (Table 3).

**Table 3:** Association between the region of origin and number of visits *p<0.05, Kruskal-Wallis was used **Bonferroni correction was applied to correct for multiple comparisons Mann-Whitney U test was used

Region of origin	Number of visits, median (Q1-Q3)	p-value	Posthoc analysis
Africa^1^	1 (1-2)	p<0,001*	P^1-2 ^=0.735
			P^1-3^=0.103
America^2^	1 (1-3)		P^1-4^=0.622
			P^1-5^<0.001**
Europe^3^	1 (1-1)		P^2-3^=0.173
			P^2-4^=0.651
Asia^4^	1 (1-2)		P^2-5^=0.145
			P^3-4^=0.147
Middle East^5^	2 (1-3)		P^3-5^<0.001**
			P^4-5^<0.001**

Analysis of the association between the region of origin and age revealed differences among all groups, but according to the posthoc analysis, the difference between the groups was not significant. Further, analysis of the association between the region of origin and clinical outcome revealed statistically significant differences between the groups (p=0.006); this difference was caused by the difference between patients from Africa and the Middle East. Among African patients, the number of patients receiving inpatient care was 25 (3.9%), whereas it was 224 (7.8%) among Middle Eastern patients. The proportion of inpatients was higher among Middle Eastern patients than among African patients (adjusted p=0.01) (Table 4).

**Table 4:** Association between the region of origin, age, and clinical outcome £ p<0.05, Kruskal-Wallis was used ¥p<0.05, Pearson Chi-squared test was used **Bonferroni correction was applied to correct for multiple comparisons Mann-Whitney U test was used

Region of origin	Age, median (Q1-Q3)	p-value		Clinical outcome Inpatient care n(%)	p-value	
Africa^1^	22 (20-23)	p=0.018^£^	P^1-2 ^=0.013	25 (3.9)	p=0.006^¥^	P^1-2 ^=0.635
			P^1-3^=0.412			P^1-3^=0.958
America^2^	28 (21-43)		P^1-4^=0.542	1 (6.3)		P^1-4^=0.451
			P^1-5^=0.068			P^1-5^=0.001**
Europe^3^	20 (2-36)		P^2-3^=0.085	1 (3.7)		P^2-3^=0.702
			P^2-4^=0.052			P^2-4^=0.819
Asia^4^	22 (19-26)		P^2-5^=0.044	15 (5)		P^2-5^=0.820
			P^3-4^=0.214			P^3-4^=0.770
Middle East^5^	21 (12-30)		P^3-5^=0.570	224 (7.8)		P^3-5^=0.430
			P^4-5^=0.028			P^4-5^=0.078

Regarding the association between clinical outcomes and the age of the patients, the median age of outpatients was 22 (17-27) years, and that of inpatients was 23 (19-30) years, with no statistically significant difference between the two groups (p=0.179) (Table 5).

**Table 5:** Association between age and clinical outcomes Mann-Whitney U test was used

Clinical outcome	Age, median (Q1-Q3)	p-value
Outpatient care	22 (17-27)	p=0.179
Inpatient care	23 (19-30)	

## Discussion

Migration movements in the last twenty years show that more than 200,000 migrants who obtain residence permits in Turkiye exist annually [2]. In 2017, Gülaçtı et al. examined Syrian refugees visiting the ED and reported that the majority of the refugees were young female patients. This finding was attributed to healthy males staying in their country of origin to fight during the war [8]. In contrast, we found that 56.6% of the patients visiting the ED were male. This difference between the findings of our and previous studies can be attributed to constant changes in the driving forces of migration, cultural characteristics of the countries of origin, and differences in access to healthcare services between the regions in which the studies were conducted.

In the present study, the most common diagnosis codes assigned to migrant patients visiting ED were "Symptoms, signs, and abnormal clinical and laboratory findings, not elsewhere classified" (R00-R99; 45.6%), followed by "Diseases of the musculoskeletal system and connective tissue" (M00-99; 29.2%), and "Diseases of the respiratory system" (J00-99; 23.1%). Studies have reported that migrant and refugee children visiting the ED frequently present with respiratory diseases and trauma [9,10]. Güngör et al. reported that the most common diagnosis codes at the visit were R00-R99 (22.63%), K00-K93 (Diseases of the digestive system) (14.61%), and J00-J99 (13.48%) [11]. Although various factors, such as language barriers in communicating with migrant patients and lack of accuracy in electronic data and medical records, prevented access to clearer diagnostic statistics, the results of the present study were found to be similar to the literature.

In the present study, most patients visiting ED were from the Middle East (74.5%), followed by Africa (16.6%). Notably, Turkiye is known to receive migration from the Middle East due to various reasons, such as war or economic issues in the neighboring countries Iraq and Syria [7,8]. However, recent changes in migration policy have also led to partnerships with countries in Africa [12]. Although many studies have examined healthcare system use and ED visits among different regions, the studies on African migrants' visits to ED in Turkiye are limited. The present study is, therefore a novel investigation.

In the present study, the proportion of students among African patients was higher. On the contrary, among Middle Eastern patients, the proportion of non-students was higher. Analysis of migration patterns and sociodemographic characteristics of migrants in Turkiye revealed that migrants of all ages from Middle East countries migrated mainly because of war and chaos in their countries of origin [12]. This may explain why the proportion of non-students was higher among Middle Eastern patients. Furthermore, Turkiye is known to host >30,000 African students from >50 African countries, and Karabuk University - the only university in Karabük province where the present study was conducted - ranks third among all Turkish universities in terms of the ratio of international students. This may be the reason why the number of students visiting ED was higher from Africa than from other regions [13,14].

We found that patients from the Middle East visited hospitals more frequently than patients from Africa. Although analysis of the association between the region of origin and age revealed differences between the groups, subgroup analyses did not reveal statistically significant differences, and there was no significant association between clinical outcomes and age. This suggests that the difference is unrelated to age but may be attributed to diagnosis or cultural characteristics.

Limitations

This was a single-center study, which may have influenced the results related to the countries of origin of the patients. In addition, studies from other cities may provide different results due to different causes of migration (tourism, health, etc.). Also, our study included a relatively young population, so further investigation is needed for the elderly.

## Conclusions

The majority of the patients who visited the ED were from the Middle East. In addition, compared to patients from other regions, patients from the Middle East had a higher number of visits and a higher likelihood of being hospitalized. Knowledge about the sociodemographic characteristics of migrant patients visiting ED and information about their diagnoses can help determine the profile of patients that emergency physicians are likely to encounter. Further studies are warranted to investigate ED visits by migrant patients in Turkiye.
